# Long-Term Effect of Febuxostat on Endothelial Function in Patients With Asymptomatic Hyperuricemia: A Sub-Analysis of the PRIZE Study

**DOI:** 10.3389/fcvm.2022.882821

**Published:** 2022-04-28

**Authors:** Tatsuya Maruhashi, Yukihito Higashi, Hisako Yoshida, Atsushi Tanaka, Kazuo Eguchi, Hirofumi Tomiyama, Kazuomi Kario, Toru Kato, Nozomu Oda, Nobuhiro Tahara, Mitsutoshi Oguri, Hirotaka Watada, Koichi Node

**Affiliations:** ^1^Department of Cardiovascular Regeneration and Medicine, Research Institute for Radiation Biology and Medicine, Hiroshima University, Hiroshima, Japan; ^2^Division of Regeneration and Medicine, Medical Center for Translational and Clinical Research, Hiroshima University Hospital, Hiroshima, Japan; ^3^Department of Medical Statistics, Osaka City University Graduate School of Medicine, Osaka, Japan; ^4^Department of Cardiovascular Medicine, Saga University, Saga, Japan; ^5^Department of General Internal Medicine, Saitama Red Cross Hospital, Saitama, Japan; ^6^Department of Cardiology, Tokyo Medical University, Tokyo, Japan; ^7^Department of Medicine, Division of Cardiovascular Medicine, Jichi Medical University School of Medicine, Shimotsuke, Japan; ^8^Department of Clinical Research, National Hospital Organization, Tochigi Medical Center, Utsunomiya, Japan; ^9^Department of Cardiology, Hiroshima Prefectural Hospital, Hiroshima, Japan; ^10^Division of Cardiovascular Medicine, Department of Medicine, Kurume University School of Medicine, Kurume, Japan; ^11^Department of Cardiology, Kasugai Municipal Hospital, Kasugai, Japan; ^12^Department of Metabolism and Endocrinology, Juntendo University Graduate School of Medicine, Tokyo, Japan

**Keywords:** xanthine oxidase, xanthine oxidase inhibitor, febuxostat, flow-mediated vasodilation, endothelial function, hyperuricemia

## Abstract

**Background:**

Xanthine oxidase is involved in the production of uric acid and the generation of superoxide anion. We evaluated the long-term effect of febuxostat, a non-purine selective xanthine oxidase inhibitor, on endothelial function in patients with asymptomatic hyperuricemia.

**Methods:**

In the PRIZE study, patients with hyperuricemia were randomly assigned to either add-on febuxostat treatment (febuxostat group) or non-pharmacologic hyperuricemia treatment (control group). Among the 514 participants, endothelial function was assessed in 41 patients in the febuxostat group and 38 patients in the control group by flow-mediated vasodilation (FMD) of the brachial artery at the beginning of the study and after 12 and/or 24 months of treatment (63 men; median age, 68.0 years).

**Results:**

The least squares mean concentration of serum uric acid was significantly lower in the febuxostat group than in the control group at 6 months (mean between-group difference [febuxostat group - control group], −2.09 mg/dL [95% confidence interval (CI), −2.520 to −1.659]; *P* < 0.001), 12 months (mean between-group difference, −2.28 mg/dL [95% CI, −2.709 to −1.842]; *P* < 0.001), and 24 months (mean between-group difference, −2.61 mg/dL [95% CI, −3.059 to −2.169]; *P* < 0.001). No significant differences were found between groups in the least squares mean estimated percentage change in FMD at 12 months (mean between-group difference, −0.56% [95% CI, −1.670 to 0.548]; *P* = 0.319) and at 24 months (mean between-group difference, −0.60% [95% CI, −1.886 to 0.685]; *P* = 0.357).

**Conclusion:**

Febuxostat treatment did not alter endothelial function assessed by FMD during a 2-year study period in patients with asymptomatic hyperuricemia.

## Introduction

Endothelial dysfunction is regarded as the initial step in the pathogenesis of atherosclerosis and plays a critical role in progression to cardiovascular complications (1, 2). In addition, endothelial function has been shown to be an independent predictor of future cardiovascular events (3, 4). Therefore, it is important to select an appropriate intervention that will effectively improve or augment endothelial function to prevent cardiovascular events in the management of patients with cardiovascular disorders.

Xanthine oxidase (XO) has been regarded as one of the major oxidase enzymes involved in the generation of reactive oxygen species (ROS) (5, 6). During purine metabolism catalyzed by XO, not only uric acid but also superoxide anion (O_2_^⋅–^) is generated concomitantly (7). Therefore, generation of ROS and production of uric acid are simultaneously increased with an increase in XO activity. ROS are involved in endothelial dysfunction by decreasing nitric oxide (NO) bioavailability through increasing NO inactivation and decreasing NO production *via* endothelial NO synthase uncoupling (2, 5). Although it remains unclear whether hyperuricemia is causally related to endothelial dysfunction in humans, experimental studies have indicated the possibility that hyperuricemia *per se* causes endothelial dysfunction through increasing inflammation or oxidative stress (8–10). Therefore, XO inhibitors have been expected to augment endothelial function by decreasing the generation of ROS and lowering serum uric acid levels (11, 12). Febuxostat is a non-purine selective XO inhibitor (13, 14). The short-term effect of febuxostat on endothelial function in humans has been investigated in a few studies (15–17). However, little information exists regarding the long-term effect of febuxostat on endothelial function in patients with asymptomatic hyperuricemia.

The PRIZE (program of vascular evaluation under uric acid control by xanthine oxidase inhibitor, febuxostat: multicenter, randomized controlled) study was a prospective, multicenter study conducted to evaluate the inhibitory effect of febuxostat on the progression of carotid artery intima-media thickness (IMT) over a 2-year follow-up period (18). In that study, flow-mediated vasodilation (FMD) of the brachial artery, an index of endothelial function, was measured in a subset of participants. Therefore, we carried out the present study as a pre-specified sub-analysis of the PRIZE study to evaluate the long-term effect of febuxostat treatment on endothelial function assessed by FMD of the brachial artery in patients with asymptomatic hyperuricemia.

## Materials and Methods

### Study Design and Patients

The rationale and design of the PRIZE study (University Hospital Medical Information Network Center: ID 000012911) have been described previously (18, 19). In brief, the PRIZE study was a multicenter, prospective, randomized, open-label and blinded-endpoint trial carried out at 48 Japanese institutions. Eligible patients were at least 20 years of age and had asymptomatic hyperuricemia with a serum uric acid level >7.0 mg/dL and a maximum IMT of the common carotid artery (CCA) ≥1.1 mm, defined as a carotid arterial plaque in the guidelines of the Japan Society of Ultrasonics in Medicine and the Japan Academy of Neurosonology (20). Patients who had taken any serum uric acid-lowering agents within the 8-week period before assessment of eligibility, those who had gouty tophus, and those who had had symptoms of gouty arthritis within 1 year before assessment of eligibility were excluded. Other exclusion criteria are described elsewhere (19).

Between May 2014 and June 2016, a total of 514 patients with asymptomatic hyperuricemia were enrolled and randomly assigned in a 1:1 ratio to either add-on febuxostat treatment (febuxostat group: *n* = 257) or non-pharmacologic hyperuricemia treatment (control group: *n* = 257). Randomization was stratified on the basis of age, sex, presence or absence of type 2 diabetes, serum uric acid level (<8.0 or ≥8.0 mg/dL), and maximum CCA-IMT (<1.3 or ≥1.3 mm) (19). Treatment of patients in the febuxostat group was initially started with febuxostat at a dose of 10 mg daily. The dose could be increased to 20 mg daily at 1 month and 40 mg daily at 2 months. Febuxostat 40 mg daily was the targeted maintenance dose. At 3 months or later, febuxostat could be further increased up to 60 mg daily. When serum uric acid levels decreased to ≤2.0 mg/dL during the study period, the maintenance dose of febuxostat was decreased by 20 mg. Participants were followed up annually for 2 years.

The primary endpoint of the PRIZE study was the percentage change in mean CCA-IMT from baseline to 24 months after treatment. Carotid ultrasound examinations were performed at the beginning of treatment and after 12 and 24 months of treatment. Exploratory endpoints included percentage changes in FMD of the brachial artery from baseline to 12 and 24 months of treatment (19). In some participating institutions, measurement of FMD of the brachial artery was optional. Among a total of 514 patients, serial measurement of FMD was performed in 41 patients in the febuxostat group and 38 patients in the control group at the beginning of the study and after 12 and/or 24 months of treatment. The data for these 79 patients from 10 institutions were analyzed in the present study. This sub-study is a pre-specified analysis (19). The study protocol was approved by the local institutional review boards and independent ethics committees at all sites. The study protocol conforms to the ethical guidelines of the 1975 Declaration of Helsinki. Written informed consent for participation in the study was obtained from all subjects.

### Study Protocol

All assessments were performed in the morning, after overnight fasting, in a quiet, dark, and air-conditioned room (constant temperature of 22 to 25°C). Subjects were kept in the supine position throughout the study. A 23-gauge polyethylene catheter was inserted into the left deep antecubital vein to obtain blood samples. The vascular response to reactive hyperemia in the brachial artery was used for the assessment of endothelium-dependent FMD. FMD measurements were performed by skilled and trained physicians or sonographers without detailed knowledge of the baseline clinical characteristics of the subjects.

### Measurement of Flow-Mediated Vasodilation

The same protocol for measurement of FMD in the brachial artery was used at all study sites. FMD was measured with the same ultrasound instrument specialized for FMD measurement in all institutions. A high-resolution linear artery transducer was coupled to computer-assisted analysis software (UNEXEF18G, UNEX Co, Nagoya, Japan) that used an automated edge detection system for measurement of brachial artery diameter. Detailed information on measurement of FMD of the brachial artery is provided in the online-only Data Supplement. In brief, FMD was measured by using a protocol in which an occlusion cuff placed around the forearm was inflated to 50 mm Hg above systolic blood pressure for 5 min to induce reactive hyperemia. Percentage of FMD [(Peak diameter - Baseline diameter)/Baseline diameter] was used for analysis (21). Intra-observer variability (coefficient of variation) was 10.1–11.2% (22).

### Statistical Analysis

All reported probability values were 2-sided, and a probability value of <0.05 was considered statistically significant. Continuous variables are summarized as mean and standard deviation for normally distributed continuous variables or median (interquartile range [IQR]) for skewed ones. The Shapiro-Wilk test was used to evaluate normality. For between-group comparisons of continuous values, Student’s *t*-test and the Wilcoxon test were used according to their respective distributions. Categorical variables are presented as frequencies and percentages and were compared by means of the χ^2^ test. We used a mixed-effects model to estimate changes in serum uric acid and percentage changes in FMD over time by treatment (febuxostat group vs. control group). To estimate group differences in serum uric acid and percentage changes in FMD, models included treatment, follow-up time, and a treatment × follow-up time interaction term. The model for percentage changes in FMD included covariates of age, sex, serum uric acid levels at each time point, and FMD at baseline. The data were processed using R 4.0.1. (R Foundation for Statistical Computing, Vienna, Austria).

## Results

### Baseline Clinical Characteristics

The baseline clinical characteristics of the subjects are summarized in Table 1. Of the 79 patients (mean age, 67.3 years; SD, 10.3 years), 63 (79.7%) were men, and 16 (20.3%) were women. Seventy-six (96.2%) had hypertension, 40 (50.6%) had dyslipidemia, 20 (25.3%) had diabetes mellitus, 11 (13.9%) were current smokers, 1 (1.3%) had previous gouty arthritis, 7 (8.9%) had previous myocardial infarction, 4 (5.1%) had stroke, and 10 (12.7%) had heart failure. There was no significant difference between the febuxostat group and the control group in any of the variables at baseline. In the febuxostat group, 11 (26.8%) patients received 10 mg, 15 (36.6%) received 20 mg, 3 (7.3%) received 30 mg, 7 (17.1%) received 40 mg, and 1 (2.4%) received 60 mg daily as the final adjusted dose of febuxostat.

**TABLE 1:** Subject clinical characteristics.

	All	Control group	Febuxostat group	
				
Variable	(*n* = 79)	(*n* = 38)	(*n* = 41)	*P*-value
Age, y (mean ± SD)	67.3 ± 10.3	67.4 ± 10.5	67.1 ± 10.2	0.898
Male, n (%)	63 (79.7)	30 (78.9)	33 (80.5)	1.000
Body mass index, kg/m^2^ (median [IQR])	25.1 (22.6–27.1)	25.5 (23.3–27.1)	24.8 (22.3–26.4)	0.298
Systolic blood pressure, mm Hg (mean ± SA)	130.4 ± 14.7	130.2 ± 15.1	130.6 ± 14.5	0.886
Diastolic blood pressure, mm Hg (mean ± SA)	75.7 ± 10.1	76.7 ± 10.9	74.7 ± 9.4	0.383
Current smoker, n (%)	11 (13.9)	2 (5.3)	9 (22.0)	0.069
**Comorbidities, n (%)**				
Hypertension	76 (96.2)	36 (94.7)	40 (97.6)	0.947
Dyslipidemia	40 (50.6)	20 (52.6)	20 (48.8)	0.907
Diabetes mellitus	20 (25.3)	11 (28.9)	9 (22.0)	0.649
Previous gouty arthritis	1 (1.3)	0 (0)	1 (2.4)	1.000
Previous myocardial infarction	7 (8.9)	3 (7.9)	4 (9.8)	1.000
Prior PCI	6 (7.6)	3 (7.9)	3 (7.3)	1.000
CABG	0 (0)	0 (0)	0 (0)	NA
Stroke	4 (5.1)	2 (5.3)	2 (4.9)	1.000
Heart failure	10 (12.7)	4 (10.5)	6 (14.6)	0.834
**Medication, n (%)**				
Antihypertensive drugs	78 (98.7)	37 (97.4)	41 (100)	0.970
ARBs	52 (65.8)	25 (65.8)	27 (65.9)	1.000
ACE inhibitors	10 (12.7)	5 (13.2)	5 (12.2)	1.000
Calcium channel blockers	57 (72.2)	27 (71.1)	30 (73.2)	1.000
β-blockers	28 (35.4)	17 (44.7)	11 (26.8)	0.100
Diuretics	23 (29.1)	12 (31.6)	11 (26.8)	0.829
Lipid-lowering drugs	34 (43.0)	16 (42.1)	18 (43.9)	1.000
Statins	33 (41.8)	15 (39.5)	18 (43.9)	0.865
Ezetimibe	4 (5.1)	1 (2.6)	3 (7.3)	0.663
Antiplatelet drugs	27 (34.2)	13 (34.2)	14 (34.1)	1.000
Aspirin	23 (29.1)	10 (26.3)	13 (31.7)	0.780

*SD indicates standard deviation; IQR, interquartile range; PCI, percutaneous coronary intervention; CABG, coronary artery bypass grafting; ARB, angiotensin receptor blocker; ACE, angiotensin-converting enzyme; NA, not applicable.*

### Serum Uric Acid Level Control

**FIGURE 1:**
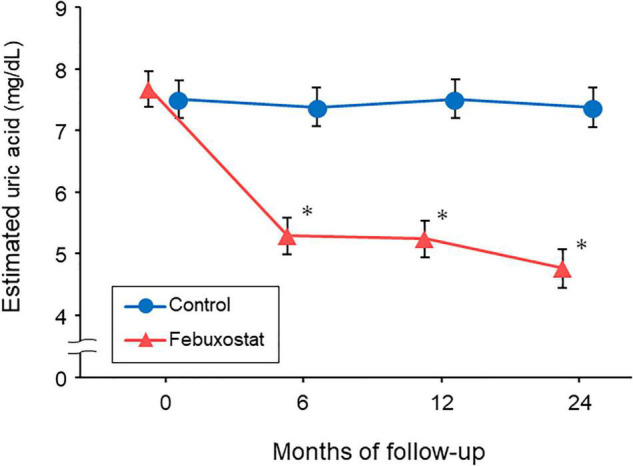
Changes in estimated serum uric acid levels in the febuxostat group and the control group. The mixed-effects model included treatment, follow-up time, and a treatment × follow-up time interaction term.^ *^P < 0.05 vs. control group at each time point.

Baseline serum uric acid levels were 7.67 mg/dL (95% confidence interval [CI], 7.38 to 7.96 mg/dL) in the febuxostat group and 7.51 mg/dL (95% CI, 7.21 to 7.82 mg/dL) in the control group. Significant differences in the serum uric acid levels were seen between the two groups at 6, 12, and 24 months (Figure 1). The least squares means of serum uric acid were lower in the febuxostat group than in the control group at 6 months (5.29 mg/dL [95% CI, 5.00 to 5.59] vs. 7.38 mg/dL [95% CI, 7.07 to 7.69]; mean between-group difference [febuxostat group - control group], −2.09 mg/dL [95% CI, −2.52 to −1.66]; *P* < 0.001), 12 months (5.24 mg/dL [95% CI, 4.94 to 5.54] vs. 7.51 mg/dL [95% CI, 7.20 to 7.83]; mean between-group difference, −2.28 mg/dL [95% CI, −2.71 to −1.84]; *P* < 0.001), and 24 months (4.76 mg/dL [95% CI, 4.46 to 5.07] vs. 7.38 mg/dL [95% CI, 7.06 to 7.70]; mean between-group difference, −2.61 mg/dL [95% CI, −3.06 to −2.17]; *P* < 0.001).

### Endothelial Function

**FIGURE 2:**
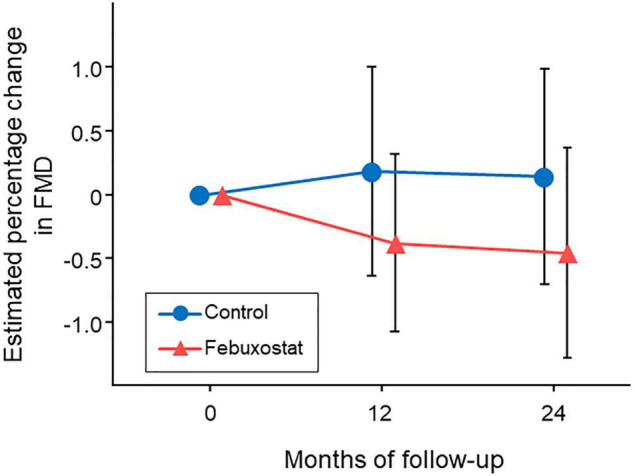
Changes in estimated percentage change in flow-mediated vasodilation (FMD) in the febuxostat group and the control group. The mixed-effects model included treatment, follow-up time, a treatment × follow-up time interaction term, age, sex, serum uric acid levels at each time point, and FMD at baseline.

Baseline FMD values were 5.34% ± 2.61% in the febuxostat group and 4.59% ± 2.73% in the control group. Estimated percentage changes in FMD from baseline at 12 and 24 months in the febuxostat group and control group are shown in Figure 2. There were no significant differences between the febuxostat group and control group in the least squares means of estimated percentage changes in FMD at 12 months (−0.38% [95% CI, −1.07 to 0.31] vs. 0.18% [95% CI, −0.64 to 1.00]; mean between-group difference, −0.56% [95% CI, −1.67 to 0.55]; *P* = 0.319) and 24 months (−0.46% [95% CI, −1.28 to 0.37] vs. 0.14% [95% CI, −0.70 to 0.98]; mean between-group difference, −0.60% [95% CI, −1.89 to 0.69]; *P* = 0.357).

**FIGURE 3:**
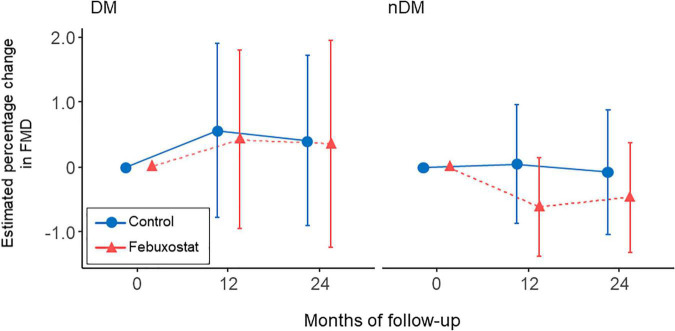
Changes in estimated percentage change in flow-mediated vasodilation (FMD) in the febuxostat group and the control group according to the presence or absence of diabetic complications. The mixed-effects model included treatment, follow-up time, a treatment × follow-up time interaction term, age, sex, serum uric acid levels at each time point, and FMD at baseline. DM indicates diabetes mellitus; nDM, non-diabetes mellitus.

Diabetic complications accounted for 20 (25.3%) of the 79 cases: 9 in the febuxostat group and 11 in the control group. We examined the interaction between the presence and absence of diabetic complications and found no interaction in the present model (p for interaction = 0.886) (Figure 3).

### Relationship Between Serum Uric Acid Levels and Flow-Mediated Vasodilation

**FIGURE 4:**
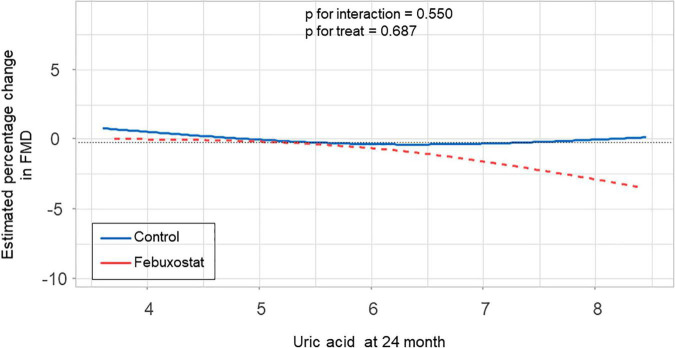
Relationship between estimated percentage change in flow-mediated vasodilation (FMD) and serum uric acid levels at 24 months in the febuxostat group and the control group adjusted for baseline FMD.

**FIGURE 5:**
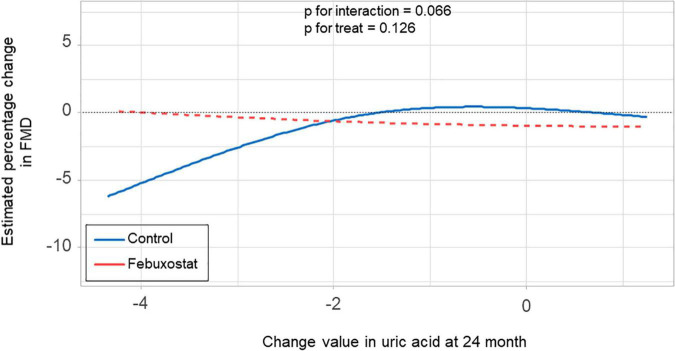
Relationship between estimated percentage change in flow-mediated vasodilation (FMD) and change in serum uric acid levels at 24 months in the febuxostat group and the control group adjusted for baseline FMD.

There was no significant difference in the relationship between estimated percentage change in FMD and serum uric acid levels at 24 months between the febuxostat group and control group (P for interaction = 0.550, P for treat = 0.687) (Figure 4). In addition, there was no significant difference in the relationship between estimated percentage change in FMD and change in serum uric acid levels at 24 months between the two groups (P for interaction = 0.066, P for treat = 0.126) (Figure 5).

## Discussion

The results of the present study demonstrated that 24 months of febuxostat treatment did not alter endothelial function assessed by FMD of the brachial artery in patients with asymptomatic hyperuricemia. To our knowledge, this is the first study in which the long-term effect of febuxostat treatment on endothelial function was investigated in patients with hyperuricemia.

NO is directly inactivated by O_2_^⋅–^ that is concomitantly generated in the process of purine metabolism catalyzed by XO. Direct reaction of NO with O_2_^⋅–^ results in the formation of peroxynitrite, a highly potent oxidant (6, 23). Tetrahydrobiopterin, an essential cofactor required for catalytic activity of endothelial NO synthase (eNOS), is oxidized to the biologically inactive form by peroxynitrite, leading to eNOS uncoupling with reduced NO formation and increased O_2_^⋅–^ production (24). Therefore, NO bioavailability is decreased by O_2_^⋅–^ generated in the process of purine metabolism catalyzed by XO through increased NO inactivation and/or decreased NO production, resulting in endothelial dysfunction. Experimental studies have indicated the possibility that uric acid *per se* causes endothelial dysfunction by being absorbed into endothelial cells through uric acid transporters and increasing inflammation or oxidative stress in endothelial cells (8, 9). Considering those putative mechanisms underlying endothelial dysfunction in patients with hyperuricemia, XO inhibitors are expected to ameliorate endothelial function through decreasing the generation of ROS and lowering serum uric acid levels in patients with hyperuricemia. Indeed, treatment with allopurinol, an XO inhibitor, has been shown clinically to improve endothelial function assessed by FMD (25, 26). Febuxostat is a non-purine selective XO inhibitor that is officially approved for treatment of patients with asymptomatic hyperuricemia in Japan. Febuxostat has been shown to have a stronger inhibitory effect than that of allopurinol on XO (27). In addition, febuxostat is expected to have antioxidative and antiatherosclerotic effects that are superior to those of allopurinol (28). Therefore, febuxostat potentially has a more beneficial effect than allopurinol on endothelial function in patients with hyperuricemia.

The short-term effect of febuxostat on endothelial function in humans has been investigated in a few studies. Tsuruta et al. reported that 4 weeks of febuxostat treatment improved endothelial function assessed by FMD in patients with hyperuricemia on hemodialysis (15), whereas Nakata et al. reported that endothelial function assessed by peripheral artery tonometry deteriorated after 3 months of febuxostat treatment in patients with hyperuricemia (16). Hays et al. reported that 6 weeks of febuxostat treatment did not improve coronary endothelial function assessed by magnetic resonance imaging in patients with stable coronary artery disease (17). Taken together, it remains controversial whether short-term febuxostat treatment ameliorates endothelial function in humans. Moreover, the long-term effect of febuxostat treatment on endothelial function remains unclear. In the present study, we showed that FMD was not improved at 12 and 24 months after febuxostat treatment in patients with asymptomatic hyperuricemia. Our findings support the main results of the PRIZE study. The PRIZE study showed that 24 months of febuxostat treatment did not delay the progression of carotid IMT in patients with asymptomatic hyperuricemia (18). These findings suggest that long-term febuxostat treatment has little antiatherosclerotic effect in patients with hyperuricemia. Although the precise reasons for the ineffectiveness of febuxostat treatment on endothelial function are unclear, one possible explanation is that the final doses of febuxostat were lower than expected. In the present study, 40 mg daily was a targeted maintenance dose of febuxostat. However, only 8 (19.5%) of 41 patients in the febuxostat group received febuxostat at ≥40 mg daily after 24 months. Therefore, we cannot exclude the possibility that the doses of febuxostat were inadequate to exert beneficial effects on endothelial function independent of the urate-lowering effect. Further studies are needed to determine whether adequate doses of febuxostat ameliorate endothelial function in patients with asymptomatic hyperuricemia.

A major limitation of the present study is the small sample size. Since this study was a sub-analysis, and FMD was a voluntary measurement parameter in the PRIZE study, the number of study subjects was relatively small. If the present results were obtained by a simple group comparison at 24 months, the power would be 20∼25% at best, and although the power is expected to be a little higher due to the mixed effects model used in this study, the number of cases is too small to be considered robust. Further studies with larger numbers of participants are needed to confirm the long-term effect of febuxostat on endothelial function in patients with asymptomatic hyperuricemia. Since most of the study’s participants were men, the results of the present study may not be generalizable to female subjects with asymptomatic hyperuricemia (29). Moreover, all of the participants were Japanese. Therefore, the results may not be generalizable to other populations. A large proportion of the patients in the present study had comorbidities such as hypertension, dyslipidemia and diabetes mellitus as well as a smoking habit and a history of cardiovascular diseases, all of which are associated with endothelial dysfunction. Although there was no statistical difference in the prevalence of those comorbidities between the febuxostat group and control group, we cannot deny the possibility that those comorbidities affected the results of the present study.

## Conclusion

In patients with asymptomatic hyperuricemia, 24 months of febuxostat treatment did not alter endothelial function. The results of the present study do not support the use of febuxostat for ameliorating endothelial function in this population.

## Data Availability Statement

The raw data supporting the conclusions of this article will be made available by the authors, without undue reservation.

## Ethics Statement

The study protocol was approved by the local institutional review boards and independent ethics committees at all sites.

## Prize Study Investigators

Principal Investigator: Koichi Node (Saga University, Saga, Japan).

PRIZE Steering Committee: Toyoaki Murohara (Nagoya University Graduate School of Medicine, Nagoya, Japan); Teruo Inoue (Dokkyo Medical University, Mibu, Japan); Masataka Sata (Tokushima University Graduate School, Tokushima, Japan); Mitsuru Ohishi (Kagoshima University, Kagoshima, Japan).

PRIZE Executive Committee: Kotaro Yokote (Chiba University Graduate School of Medicine, Chiba, Japan); Kazuomi Kario (Jichi Medical University School of Medicine, Shimotsuke, Japan), Hirotaka Watada (Juntendo University Graduate School of Medicine, Tokyo, Japan); Iichiro Shimomura (Osaka University, Graduate School of Medicine, Suita, Japan); Munehide Matsuhisa (Tokushima University Graduate School, Tokushima, Japan); Yoshihiro Fukumoto (Kurume University School of Medicine, Kurume, Japan); Koji Maemura (Nagasaki University Graduate School of Biomedical Sciences, Nagasaki, Japan); Yusuke Ohya (University of the Ryukyus, Okinawa, Japan).

PRIZE Site Investigators: Yuichi Akasaki (Kagoshima University, Kagoshima, Japan); Junya Ako (Kitasato University School of Medicine, Sagamihara, Japan); Hirohisa Amano (Dokkyo Medical University, Mibu, Japan); Kazutaka Aonuma (Graduate School of Comprehensive Human Sciences, University of Tsukuba, Tsukuba, Japan); Yutaka Aoyama (Nagoya Daini Red Cross Hospital, Nagoya, Japan); Hirofumi Arai (Kameda Medical Center, Komogawa, Japan); Kuniya Asai (Nippon Medical School, Tokyo, Japan); Machiko Asaka (Saga University, Saga, Japan); Yoshifumi Awaji (Nagoya Ekisaikai Hospital, Nagoya, Japan); Noriko Ban (Chiba Aoba Municipal Hospital, Chiba, Japan); Toshiaki Ban (Isumi Medical Center, Isumi, Japan); Yasuko K Bando (Nagoya University Graduate School of Medicine, Nagoya, Japan); Hiroyuki Daida (Juntendo University Graduate School of Medicine, Tokyo, Japan); Shunsuke Eguchi (Japanese Red Cross Nagoya Daini Hospital, Nagoya, Japan); Mami Enomoto (Graduate School of Comprehensive Human Sciences, University of Tsukuba, Tsukuba, Japan); Yuichi Fujii (Hiroshima General Hospital of West Japan Railway Company, Hiroshima, Japan); Akinori Fujikake (Dokkyo Medical University Saitama Medical Center, Koshigaya, Japan); Masanori Fujimoto (Graduate School of Medicine, Chiba University, Chiba, Japan); Tomohiro Fujisaka (Osaka Medical College, Takatsuki, Japan); Shuichi Fujita (Osaka Medical College, Takatsuki, Japan); Satoki Fukae (Nagasaki University Graduate School of Biomedical Sciences, Nagasaki, Japan); Daiju Fukuda (Tokushima University Graduate School of Biomedical Sciences, Tokushima, Japan); Mieko Fukui (Kimitsu Chuo Hospital, Kisarazu, Japan); Yuhei Goriki (Miyazaki Medical Association Hospital, Miyazaki, Japan); Shuichi Hamasaki (Kagoshima City Hospital, Kagoshima, Japan); Tomoya Hara (Tokushima University Graduate School of Biomedical Sciences, Tokushima, Japan); Hiroshi Hasegawa (Chiba University Graduate School of Medicine, Chiba, Japan); Kenichi Hashimoto (National Defense Medical College, Tokorozawa, Japan); Mitsumasa Hata (Sekino Hospital, Tokyo, Japan); Shiro Hata (Sasebo City General Hospital, Sasebo, Japan); Ryo Hayashida (Nagoya University Graduate School of Medicine, Nagoya, Japan); Akihiro Higashi (Dokkyo Medical University Saitama Medical Center, Koshigaya, Japan); Seiichiro Higuchi (Graduate School of Medicine, Chiba University, Chiba, Japan); Akihiro Honda (Kurume University School of Medicine, Kurume, Japan); Satoshi Hoshide (Jichi Medical University School of Medicine, Shimotsuke, Japan); Masaaki Hoshiga (Osaka Medical College, Takatsuki, Japan); Junko Hotchi (Tokushima University Graduate School of Biomedical Sciences, Tokushima, Japan); Sachiyo Igata (Kurume University School of Medicine, Kurume, Japan); Yumi Ikehara (University of the Ryukyus, Nishihara, Japan); Teruo Inoue (Dokkyo Medical University, Mibu, Japan); Youhei Inoue (Miyazaki Medical Association Hospital, Miyazaki, Japan); Hiroko Ishigami (Nagoya Daini Red Cross Hospital, Nagoya, Japan); Masaharu Ishihara (Hyogo College of Medicine, Nishinomiya, Japan); Hideki Ishii (Nagoya University Graduate School of Medicine, Nagoya, Japan); Tetsuya Ishikawa (Dokkyo Medical University Saitama Medical Center, Koshigaya, Japan); Takashi Ishimatsu (Nagasaki University Graduate School of Biomedical Sciences, Nagasaki, Japan); Yusuke Ishiyama (Jichi Medical University School of Medicine, Shimotsuke, Japan); Takahide Ito (Osaka Medical College, Takatsuki, Japan); Ayumi Ito (Nagoya Daini Red Cross Hospital, Nagoya, Japan); Toshiaki Kadokami (Fukuoka Saiseikai Futsukaichi Hospital, Chikushino, Japan); Haruo Kamiya (Japanese Red Cross Nagoya Daiichi Hospital, Nagoya, Japan); Soichiro Kashihara (Fukuoka Saiseikai Futsukaichi Hospital, Chikushino, Japan); Yoshihiro Kawamura (Kasugai Municipal Hospital, Kasugai, Japan); Kazuo Kitagawa (Tokyo Women’s Medical University, Tokyo, Japan); Yoshio Kobayashi (Chiba University Graduate School of Medicine, Chiba, Japan); Satoshi Kodera (Asahi General Hospital, Asahi, Japan); Seiji Koga (Nagasaki University Graduate School of Biomedical Sciences, Nagoya, Japan); Hisashi Koide (Chiba University Graduate School of Medicine, Chiba, Japan); Yuji Koide (Nagasaki University Graduate School of Biomedical Sciences, Nagasaki, Japan); Hiroshi Koiwaya (Miyazaki Medical Association Hospital, Miyazaki, Japan); Hiroki Kojima (Nagoya University Graduate School of Medicine, Nagoya, Japan); Eri Komai (Graduate School of Medicine, Chiba University, Chiba, Japan); Takaaki Komatsu (Dokkyo Medical University Saitama Medical Center, Koshigaya, Japan); Shingo Kono (Kobe City Medical Center General Hospital, Kobe, Japan); Takashi Kono (Graduate School of Medicine, Chiba University, Chiba, Japan); Yoshiaki Kubota (Nippon Medical School, Tokyo, Japan); Akio Kuroda (Institute of Advanced Medical Sciences, Tokushima University, Tokushima, Japan); Takanori Kuroyanagi (Dokkyo Medical University Saitama Medical Center, Koshigaya, Japan); Akifumi Kushiyama (The Institute for Adult Diseases, Asahi Life Foundation, Tokyo, Japan); Kenya Kusunose (Tokushima University Graduate School of Biomedical Sciences, Tokushima, Japan); Tatsuya Maruhashi (Graduate School of Biomedical and Health Sciences, Hiroshima University, Hiroshima, Japan); Kazuo Matsunaga (Imari Arita Kyoritsu Hospital, Matsuura, Japan); Tomomi Matsuura (Tokushima University Graduate School of Biomedical Sciences, Tokushima, Japan); Takafumi Mayama (Graduate School of Medicine, Chiba University, Chiba, Japan); Daigo Mine (Saga-Ken Medical Centre Koseikan, Saga, Japan); Masatoshi Miyamura (Osaka Medical College, Takatsuki, Japan); Ryota Morimoto (Nagoya University Graduate School of Medicine, Nagoya, Japan); Hideaki Morita (Osaka Medical College, Takatsuki, Japan); Hidekazu Nagano (Chiba University Graduate School of Medicine, Chiba, Japan); Hidemitsu Nakagawa (Nozaki Tokushukai Hospital, Daito, Japan); Katsunori Nakamura (Ryukyu University Hospital, Nishihara, Japan); Ryo Nakamura (Fukuoka Saiseikai Futsukaichi Hospital, Chikushino, Japan); Ikuko Nakamura (Saga-Ken Medical Centre Koseikan, Saga, Japan); Hitoshi Nakashima (National Hospital Organization Kagoshima Medical Center, Kagoshima, Japan); Mamoru Nanasato (Japanese Red Cross Nagoya Daini Hospital, Nagoya, Japan); Isao Nishi (National Hospital Organization Kasumigaura Medical Center, Tsuchiura, Japan); Shinichi Niwano (Kitasato University School of Medicine, Sagamihara, Japan); Shuichi Nomura (Hiroshima General Hospital of West Japan Railway Company, Hiroshima, Japan); Nozomu Oda (Graduate School of Biomedical and Health Sciences, Hiroshima University, Hiroshima, Japan); Shio Oguchi (Kasugai Municipal Hospital, Kasugai, Japan); Mitsutoshi Oguri (Kasugai Municipal Hospital, Kasugai, Japan); Arihide Okahara (Saga-Ken Medical Centre Koseikan, Saga, Japan); Masaaki Okutsu (Nozaki Tokushukai Hospital, Daito, Japan); Fumitake Ozaki (Dokkyo Medical University Saitama Medical Center, Koshigaya, Japan); Michishige Ozeki (Osaka Medical College, Takatsuki, Japan); Tomoko Saisu (Tokyo Medical University, Tokyo, Japan); Yuichi Saito (Chiba University Hospital, Chiba, Japan); Makoto Saitoh (Nishio Municipal Hospital, Nishio, Japan); Yosuke Saka (Kasugai Municipal Hospital, Kasugai, Japan); Yoshihiko Sakai (Dokkyo Medical University Saitama Medical Center, Koshigaya, Japan); Kazushi Sakane (Osaka Medical College, Takatsuki, Japan); Ikki Sakuma (Graduate School of Medicine, Chiba University, Chiba, Japan); Shakya Sandeep (Asahi General Hospital, Asahi, Japan); Hiroaki Sano (Nagoya Ekisaikai Hospital, Nagoya, Japan); Hisakuni Sekino (Sekino Hospital, Tokyo, Japan); Yuka Senoo (Nagoya Daini Red Cross Hospital, Nagoya, Japan); Kensaku Shibata (Osaka Medical College, Takatsuki, Japan); Yoshisato Shibata (Miyazaki Medical Association Hospital, Miyazaki, Japan); Takahisa Shibata (Isumi Medical Center, Isumi, Japan); Akina Shiga (Graduate School of Medicine, Chiba University, Chiba, Japan); Kazuki Shiina (Tokyo Medical University, Tokyo, Japan); Michio Shimabukuro (Tokushima University Graduate School of Biomedical Sciences, Tokushima, Japan); Yusaku Shimbo (Nagoya University Graduate School of Medicine, Nagoya, Japan); Wataru Shimizu (Nippon Medical School, Tokyo, Japan); Masahisa Shimpo (Jichi Medical University School of Medicine, Shimotsuke, Japan); Takeshi Soeki (Tokushima University Graduate School of Biomedical Sciences, Tokushima, Japan); Koichi Sohmiya (Osaka Medical College, Takatsuki, Japan); Hiroyuki Suzuki (Nagoya Daini Red Cross Hospital, Nagoya, Japan); Susumu Suzuki (Nagoya University Graduate School of Medicine, Nagoya, Japan); Makoto Suzuki (Kameda Medical Center, Kamogawa, Japan); Nobuhiro Tahara (Kurume University School of Medicine, Kurume, Japan); Tazu Tahara (The Institute for Adult Diseases, Asahi Life Foundation, Tokyo, Japan); Sadako Takahashi (Jichi Medical University School of Medicine, Shimotsuke, Japan); Bonpei Takase (National Defense Medical College, Tokorozawa, Japan); Kaoru Takegami (Saga-Ken Medical Centre Koseikan, Saga, Japan); Tomoko Takiguchi (Kimitsu Chuo Hospital, Kisarazu, Japan); Tomonobu Takikawa (Kasugai Municipal Hospital, Kasugai, Japan); Ai Tamura (Graduate School of Medicine, Chiba University, Chiba, Japan); Tomoaki Tanaka (Chiba University Graduate School of Medicine, Chiba, Japan); Akihito Tanaka (Nagoya University Graduate School of Medicine, Nagoya, Japan); Hiroyuki Tanaka (Niko Clinic, Takeo, Japan); Jun Tanigawa (Osaka Medical College, Takatsuki, Japan); Daisuke Tanimura (Nagoya Ekisaikai Hospital, Nagoya, Japan); Yosuke Tatami (Nagoya University Graduate School of Medicine, Nagoya, Japan); Takashi Terano (Chiba Aoba Municipal Hospital, Chiba, Japan); Fumio Terasaki (Osaka Medical College, Takatsuki, Japan); Tomoyuki Tobushi (Fukuoka Saiseikai Futsukaichi Hospital, Chikushino, Japan); Seiko Tokoi (Dokkyo Medical University, Mibu, Japan); Toshiyuki Tsubouchi (Nozaki Tokushukai Hospital, Daito, Japan); Daigaku Uchida (Hotaruno Central Clinic, Kisarazu, Japan); Tomohiro Ueda (Hiroshima General Hospital of West Japan Railway Company, Hiroshima, Japan); Rie Ueno (Tokushima University Graduate School of Biomedical Sciences, Tokushima, Japan); Hiromi Ueno (Jichi Medical University School of Medicine, Shimotsuke, Japan); Chikara Ueyama (Gifu Prefectural Tajimi Hospital, Tajimi, Japan); Tetsuzo Wakatsuki (Tokushima University Graduate School of Biomedical Sciences, Tokushima, Japan); Tomohiko Watanabe (Osaka Medical College, Takatsuki, Japan); Masato Watarai (Anjo Kosei Hospital, Anjo, Japan); Isao Yaguchi (Dokkyo Medical University Saitama Medical Center, Koshigaya, Japan); Ayumu Yajima (Saga University, Saga, Japan); Jiko Yamada (Tokyo Medical University, Tokyo, Japan); Kyohei Yamamoto (Chiba Aoba Municipal Hospital, Chiba, Japan); Sachiko Yamauchi (Ryukyu University Hospital, Nishihara, Japan); Yohei Yamauchi (Osaka Medical College, Takatsuki, Japan); Naoto Yokota (Yokota Naika, Miyazaki, Japan); Tomohiko Yoshida (Chiba Aoba Municipal Hospital, Chiba, Japan); Goro Yoshioka (Miyazaki Medical Association Hospital, Miyazaki, Japan).

Members of the Data and Safety Monitoring Board: Hiroyuki Daida (Juntendo University Graduate School of Medicine, Tokyo, Japan); Junya Ako (Kitasato University School of Medicine, Sagamihara, Japan); Kazuo Kitagawa (Tokyo Women’s Medical University, Tokyo, Japan).

Members of the Clinical Events Committee: Wataru Shimizu (Nippon Medical School, Tokyo, Japan); Yoshio Kobayashi (Chiba University Graduate School of Medicine, Chiba, Japan); Masaharu Ishihara (Hyogo College of Medicine, Nishinomiya, Japan).

Imaging Core Laboratory: Tsukuba Echo Core Laboratory. LLC; Tomoko Ishizu (Tsukuba University, Tsukuba, Japan).

Monitoring: Shinichiro Ueda (Clinical Research Management Center, University of the Ryukyus, Okinawa, Japan).

Audit Team: Clinical Research Support Center, University of the Ryukyus, Okinawa, Japan.

Trial Secretariat: Atsushi Tanaka (Saga University, Saga, Japan); Jun-ichi Oyama (Saga University, Saga, Japan); Mikiko Kagiyama (Saga University, Saga, Japan); Nouvelle Place Inc., Tokyo, Japan; Organization for Clinical Medicine Promotion, Tokyo, Japan.

## Author Contributions

YH and TM drafted the article and conception of this study. HY performed the statistical analysis. KE, HT, KK, TK, NO, NT, MO, and HW measured the FMD. AT and KN revised the article critically for important intellectual content. All authors contributed to the article and approved the submitted version.

## Conflict of Interest

YH received honoraria and grants from Teijin, Boehringer Ingelheim, MSD, Sanofi, AstraZeneca, Kyowa Hakko Kirin, Takeda, Astellas, Daiichi Sankyo, Mochida, Nihon Kohden, Shionogi, Nippon Sigmax, Sanwa Kagaku Kenkyusho, Unex, and Kao; honoraria from Radiometer, Omron, Sumitomo Dainippon, Otsuka, Torii, Kowa, Fujiyakuhin, Amgen, Nippon Shinyaku, Itamar, Bayer, Eli Lilly, and Ono. AT received honoraria from Boehringer Ingelheim and research funding from GlaxoSmithKline. HT received funds from Omron Health Care Company, Asahi Calpis Wellness Company and Teijin Pharma Company. HW received honoraria for lectures for Mitsubishi Tanabe Pharma, Dainippon Sumitomo Pharma, Sanwa Kagaku, Takeda, Sanofi, Kowa, Merck Sharp; and Dohme, Boehringer Ingelheim, Eli Lilly, and Novo Nordisk, and research activities for Takeda, Boehringer Ingelheim, Kissei Pharma, Novo Nordisk, Mitsubishi Tanabe Pharma, Lifescan Japan, Dainippon Sumitomo Pharma, Kyowa Kirin, and Merck Sharp; and Dohme. KN received research grants from Asahi Kasei, Astellas, Bayer, Boehringer Ingelheim, Mitsubishi Tanabe, Teijin, and Terumo; scholarships from Astellas, Bayer, Bristol-Myers Squibb, Daiichi Sankyo, Daiichi Sankyo Healthcare, Takeda, and Teijin; and personal fees from Astellas, AstraZeneca, Bayer, Boehringer Ingelheim, Daiichi Sankyo Healthcare, Eli Lilly, Kowa, Mitsubishi Tanabe, MSD, Novartis, Ono, Takeda, and Teijin. The remaining authors declare that the research was conducted in the absence of any commercial or financial relationships that could be construed as a potential conflict of interest.

## Publisher’s Note

All claims expressed in this article are solely those of the authors and do not necessarily represent those of their affiliated organizations, or those of the publisher, the editors and the reviewers. Any product that may be evaluated in this article, or claim that may be made by its manufacturer, is not guaranteed or endorsed by the publisher.
