# Supplementary material for: Long-Term Effect of Febuxostat on Endothelial Function in Patients With Asymptomatic Hyperuricemia: A Sub-Analysis of the PRIZE Study
Source: Front Cardiovasc Med. 2022 Apr 28;9:882821. doi: 10.3389/fcvm.2022.882821 (PMC9095910; doi:10.3389/fcvm.2022.882821)
Supplement: Supplementary file 1 [file Presentation_1.pdf]

SUPPLEMENTAL FILE

Long-term Effect of Febuxostat on Endothelial Function in Patients with Asymptomatic Hyperuricemia: A Sub-analysis of the PRIZE Study

Brief title: Febuxostat and Endothelial Function

Tatsuya Maruhashi, MD, PhD[1], Yukihito Higashi, MD, PhD[1,2], Hisako Yoshida, PhD[3], Atsushi Tanaka, MD, PhD[4], Kazuo Eguchi, MD, PhD[5], Hirofumi Tomiyama, MD, PhD[6], Kazuomi Kario, MD, PhD[7], Toru Kato, MD, PhD[8], Nozomu, Oda, MD, PhD[9], Nobuhiro Tahara, MD, PhD[10], Mitsutoshi Oguri, MD, PhD[11], Hirotaka Watada, MD, PhD[12], and Koichi Node, MD, PhD[4], for the PRIZE Study Investigators

[1] Department of Cardiovascular Regeneration and Medicine, Research Institute for Radiation Biology and Medicine, Hiroshima University, Hiroshima, Japan
[2] Division of Regeneration and Medicine, Medical Center for Translational and Clinical Research, Hiroshima University Hospital, Hiroshima, Japan
[3] Department of Medical Statistics, Osaka City University Graduate School of Medicine, Osaka, Japan
[4] Department of Cardiovascular Medicine, Saga University, Saga, Japan
[5] Department of General Internal Medicine, Saitama Red Cross Hospital, Saitama, Japan
[6] Department of Cardiology, Tokyo Medical University, Tokyo, Japan
[7] Division of Cardiovascular Medicine, Department of Medicine, Jichi Medical University School of Medicine, Shimotsuke, Japan
[8] Department of Clinical Research, National Hospital Organization, Tochigi Medical Center, Utsunomiya, Japan
[9] Department of Cardiology, Hiroshima Prefectural Hospital, Hiroshima, Japan
[10] Division of Cardiovascular Medicine, Department of Medicine, Kurume University School of Medicine, Kurume, Japan
[11] Department of Cardiology, Kasugai Municipal Hospital, Kasugai, Japan
[12] Department of Metabolism and Endocrinology, Juntendo University Graduate School of Medicine, Tokyo, Japan

Address for correspondence: Yukihito Higashi, MD, PhD, FAHA
Department of Cardiovascular Regeneration and Medicine,
Research Institute for Radiation Biology and Medicine, Hiroshima University
1-2-3 Kasumi, Minami-ku, Hiroshima 734-8551, Japan
Phone: +81-82-257-5831 Fax: +81-82-257-5831
E-mail: yhigashi@hiroshima-u.ac.jp

Methods

Measurements of Flow-mediated Vasodilation (FMD) of the brachial artery

Vascular response to reactive hyperemia in the brachial artery was used for assessment of endothelium-dependent FMD. A high-resolution linear artery transducer was coupled to computer-assisted analysis software (UNEXEF18G, UNEX Co, Nagoya, Japan) that used an automated edge detection system for measurement of brachial artery diameter. A blood pressure cuff was placed around the forearm. The brachial artery was scanned longitudinally 5 to 10 cm above the elbow. When the clearest B-mode image of the anterior and posterior intimal interfaces between the lumen and vessel wall was obtained, the transducer was held at the same point throughout the scan by a special probe holder (UNEX Co) to ensure consistency of the image. Depth and gain setting were set to optimize the images of the arterial lumen wall interface. When the tracking gate was placed on the intima, the artery diameter was automatically tracked, and the waveform of diameter changes over the cardiac cycle was displayed in real time using the FMD mode of the tracking system. This allowed the ultrasound images to be optimized at the start of the scan and the transducer position to be adjusted immediately for optimal tracking performance throughout the scan. Pulsed Doppler flow was assessed at baseline and during peak hyperemic flow, which was confirmed to occur within 15 s after cuff deflation. Blood flow velocity was calculated from the color Doppler data and was displayed as a waveform in real time. The baseline longitudinal image of the artery was acquired for 30 s, and then the blood pressure cuff was inflated to 50 mm Hg above systolic pressure for 5 min.[1] The longitudinal image of the artery was recorded continuously until 5 min after cuff deflation. Pulsed Doppler velocity signals were obtained for 20 s at baseline and for 10 s immediately after cuff deflation. Changes in brachial artery diameter were immediately expressed as percentage change relative to the vessel diameter before cuff inflation. FMD was automatically calculated as the percentage change in peak vessel diameter from the baseline value. Percentage of FMD [(Peak diameter - Baseline diameter)/Baseline diameter] was used for analysis. Blood flow volume was calculated by multiplying the Doppler flow velocity (corrected for the angle) by heart rate and vessel cross-sectional area ($-r^2$). Reactive hyperemia was calculated as the maximum percentage increase in flow after cuff deflation compared with baseline flow.
